# Optimal neighborhood indexing for protein similarity search

**DOI:** 10.1186/1471-2105-9-534

**Published:** 2008-12-16

**Authors:** Pierre Peterlongo, Laurent Noé, Dominique Lavenier, Van Hoa Nguyen, Gregory Kucherov, Mathieu Giraud

**Affiliations:** 1 Symbiose team-project, IRISA INRIA, Rennes, France; 2 LIFL, CNRS, Université Lille 1, Lille, France; 3 INRIA Lille Nord-Europe, Lille, France; 4 IRISA – ENS Cachan, France

## Abstract

**Background:**

Similarity inference, one of the main bioinformatics tasks, has to face an exponential growth of the biological data. A classical approach used to cope with this data flow involves heuristics with large seed indexes. In order to speed up this technique, the index can be enhanced by storing additional information to limit the number of random memory accesses. However, this improvement leads to a larger index that may become a bottleneck. In the case of protein similarity search, we propose to decrease the index size by reducing the amino acid alphabet.

**Results:**

The paper presents two main contributions. First, we show that an optimal neighborhood indexing combining an alphabet reduction and a longer neighborhood leads to a reduction of 35% of memory involved into the process, without sacrificing the quality of results nor the computational time. Second, our approach led us to develop a new kind of substitution score matrices and their associated *e*-value parameters. In contrast to usual matrices, these matrices are rectangular since they compare amino acid groups from different alphabets. We describe the method used for computing those matrices and we provide some typical examples that can be used in such comparisons. Supplementary data can be found on the website .

**Conclusion:**

We propose a practical index size reduction of the neighborhood data, that does not negatively affect the performance of large-scale search in protein sequences. Such an index can be used in any study involving large protein data. Moreover, rectangular substitution score matrices and their associated statistical parameters can have applications in any study involving an alphabet reduction.

## Background

One fundamental task in bioinformatics concerns large scale comparisons between proteins or families of proteins. It often constitutes the first step before further investigations. A typical comparison, for example, is to query a database with a newly discovered sequence. Observed similarities witness a putative common biological function and direct further studies.

In this paper, we focus on massive protein sequence comparisons: a large database is iteratively compared with relatively short queries (such as newly sequenced data). A possible approach is to use the exact dynamic programming method [1]. For a given similarity model, this method provides optimal alignments within a quadratic computation time. Some optimizations achieve a sub-quadratic complexity [2], but the computation time remains prohibitive for large scale comparisons. Thus, in practice, the full dynamic programming approach is applied to comparison of short sequences.

A successful family of similarity search methods is provided by *seed-based heuristics*, starting with Fasta [3] and Blast [4] and including specific methods for protein similarities such as Blastp [5]. Seed-based heuristics were recently enhanced by advanced seeding tools like the *spaced seeds *used in PatternHunter [6] or Yass [7] (see [8] for a recent survey). Authors of this paper also worked on the alliance between advanced seeds techniques and reconfigurable architectures [9].

**Figure 1:**
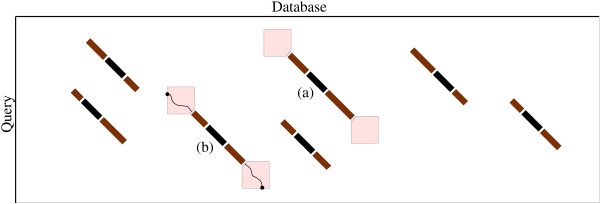
**Schematic view of a Blast-like 3-stage algorithm**. Representation of the three stages of comparison of a query (vertical) against a database (horizontal): Stage 1: identify seeds, i.e. small patterns occurring in both the query and the database (black diagonals). Stage 2: compute seed extensions and keep only those for which the score verifies at threshold T (brown diagonals). On the Figure, seeds (*a*) and (*b*) are successfully extended. Stage 3: perform a full dynamic programming computation (white squares) on remaining seeds. In this example, only seed (*b*) leads to a significant alignment.

The main idea of seed-based heuristics is to anchor the detection of similarities using matching short words or short subsequences occurring in both compared sequences. The form of these words or subsequences is provided by a pattern called a *seed*. A word that respects the seed is called a *key*. For instance, MVK is one of 20^3 ^possible keys for the seed of three consecutive characters on the protein alphabet. Detection of similarities between two strings is done in three stages, as presented in Figure 1:

• Stage 1: search for keys that occur in both strings,

• Stage 2: extension of these matching keys with an ungapped alignment, keeping only the alignments with a score greater than a given threshold T,

• Stage 3: full dynamic programming algorithm, applied only to successfully extended matching keys.

In this work, we consider comparisons between a set of protein queries against a large protein database of *N *amino acids. A common usage of Blast is to index the queries, and then to scan the full database at the runtime. If the size of the query and the database allow it, a full indexation of both leads to advantageous results [10]. In our work, we applied approach used e.g. in Blat [11] where the database is indexed once and each query is successively processed.

**Figure 2:**
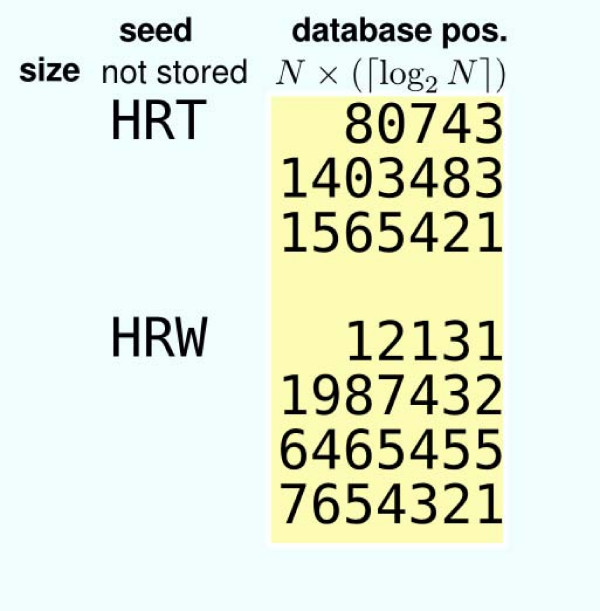
**Offset indexing**. Fragment of an offset index. For each seed key (here composed of three letters), the list of its occurrence positions is stored.

To be efficient, the database positions are indexed by seed keys. The usual indexing scheme is shown Figure 2: for each key, a list of all its occurrences is stored. At Stage 1, each query position corresponds to a seed key (or, for the Blastp approach, a set of seed keys that are similar to the query seed key). An index access provides the list of key occurrences in the database, enabling Stage 2. We call such an approach the offset indexing approach. In this case, for each seed position, an offset of ⌈log_2 _*N*⌉ bits is stored. The index size is thus equal to *S*_offset _= *N *× ⌈log_2 _*N*⌉ bits.

**Figure 3:**
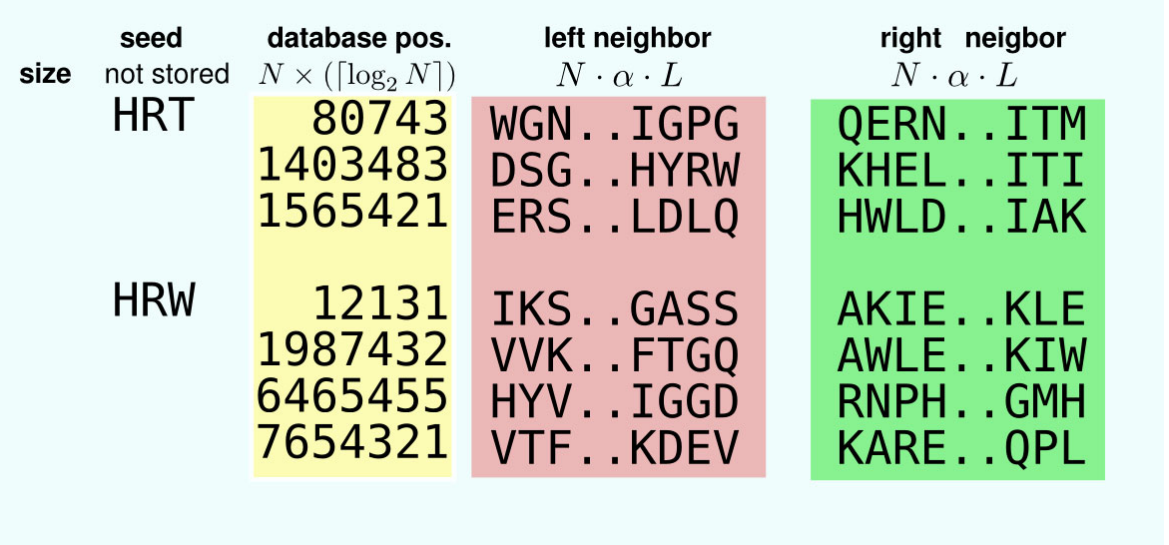
**Neighborhood indexing**. Fragment of a neighborhood index. For each seed key, the list of its occurrence positions is stored. For each occurrence, its right and left neighborhoods are additionally stored.

For each query position, each execution of Stage 2 needs to access all the occurrences of the corresponding key. This leads to numerous random memory accesses that are time consuming: memory accesses at random positions are not efficiently cached and require high latencies [12]. A way to reduce the computation time is thus to avoid as far as possible such random memory accesses. For that purpose, it is possible to additionally store, for each key occurrence, its left and right neighborhoods in the sequence, as illustrated in Figure 3. Thus, given a position in the query and its corresponding key, all neighborhoods of this key occurrences in the database are obtained through a single random memory access. For each database position, two neighborhoods are additionally stored. We call this indexing approach the neighborhood indexing approach. The overall index size is then equal to *S*_neighborhood _= *N *× (⌈log_2 _*N*⌉ + 2*αL*) bits, where

• *α *is the number of bits for coding a character (amino acid), and

• *L *is the length of each neighborhood.

**Figure 4:**
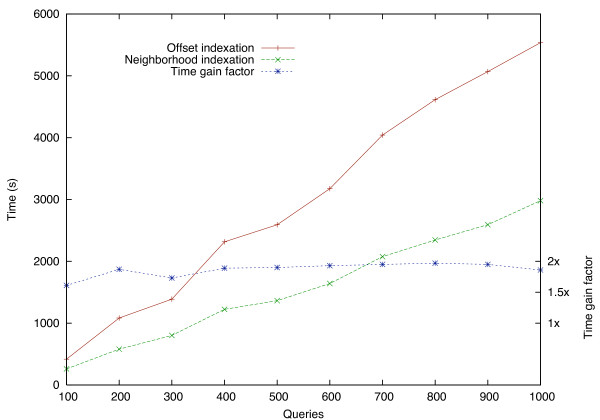
**Time saved by neighborhood indexing compared to offset indexing**. Execution time using the offset indexing and the neighborhood indexing for comparing a growing set of queries against a bank of 70·10^3 ^proteins.

As seen in Figure 4, the main advantage of the neighborhood indexing is that it speeds up the execution time by a factor ranging between 1.5 and 2 over the offset indexing. The actual speed gain depends on the database length and on many implementation and architecture parameters (such as memory and cache sizes, cache strategies and access times) that will not be discussed here. An obvious drawback of the neighborhood indexing is the additional memory it requires to store neighborhoods. Comparing the two indexing schemes, the ratio *r *between the overall index sizes of the neighborhood indexing and the offset indexing is

$$r=\frac{S_{\text{neighborhood}}}{S_{\text{offset}}}=1+\frac{2\alpha L}{\left\lceil \log_{2}N\right\rceil }.$$

In common experiments, ⌈log_2 _*N*⌉ is between 20 and 40, *αL *is between 20 and 200, hence *r *is between 2 and 21. It is worth mentioning that the ⌈log_2 _*N*⌉ value is often raised to a more practical 32 or 64 bits, reducing the ratio *r *even more. Storing neighborhoods becomes then relevant with the reduction of memory prices. For instance, the modern technology brings the possibility to get gigabytes of Flash memory in a personal computer for some hundred dollars. It is thus interesting to exploit this storage space as much as possible. It can be used for treating larger databases, but also, as in this work, for speeding up widely used applications.

However, the index size still remains the main limitation. In this paper, we study how the size of a large neighborhood index can be reduced while preserving the result quality. For this purpose, we worked on reducing as much as possible the ratio *r*. A way for doing this is to reduce the factor *αL*. We propose to simultaneously increase the neighborhood length (*L*) and reduce the alphabet size (2·*α*). We limit the alphabet size by partitioning amino acids into groups. This reduces *a *by encoding neighborhood characters in less than 5 bits required for coding 20 amino acids. Partitioning the amino acids into 16 groups enables to encode each group using 4 bits, and partitioning into 8, 4 or 2 groups enables to encode each group by 3, 2, and 1 bits respectively. All these reduced alphabets are tested in this paper.

Grouping amino acids was studied in several papers [13-16]. Groups can rely on amino acid physical-chemical properties or on a statistical analysis of alignments. For example, the authors of [13] computed correlation coefficients between pairs of amino acids based on the BLOSUM50 matrix and used a greedy algorithm to merge them. A branch-and-bound algorithm for partitioning the amino acids was proposed in [14]. Those papers mainly deal with the construction of reduced alphabets, but none of them studies how the alphabet reduction affects the sensitivity of similarity search, or undertakes a quantitative analysis of the trade-off between search sensitivity and index size for those alphabets. This raises the following problem that is solved in this paper: *Can reduced alphabets allow one to decrease the factor αL while preserving the quality of similarity search results?*

## Results and discussion

The main result of our work is an effective reduction of the index size without deteriorating the quality of the results of similarity search. Moreover, we provide substitution score matrices and *e*-value parameters to be used with reduced alphabets. Our results are based on the alphabets defined by the amino acids groups proposed by Li and al. (Table 2 of [15]). This choice was motivated by empirical tests showing their relevancy with seeds matching. However, our method can be applied to any other amino acids partitions. The website [17] provides data for all the alphabets reported in [16].

**Table 1:** Stage two algorithm

**Algorithm 1 **Stage 2
**Ensure**: reports if a matching key occurrence potentially belongs to an alignment
**Require**: query neighborhoods (left_query _and right_query_)
1: get database neighborhoods left_db _and right_db_
2: *result*_left _← 0; *highest*_left _← 0
3: *result*_right _← 0; *highest*_right _← 0
4: **for ***i *from 1 to *L ***do**
5: *result*_left _← *result*_left _+ *subst*_*score *(left_db _[*i*], left_query _[*i*])
6: **if ***result*_left _> *highest*_left _**then ***highest*_left _← *result*_left _**endif**
7: **end for**
8: **for ***i *from 1 to *L ***do**
9: *result*_right _← *result*_right _+ *subst*_*score*(right_db _[*i*], right_query _[*i*])
10: **if ***result*_right _> *highest*_right _**then ***highest*_right _← *result*_right _**endif**
11: **end for**
12: **if ***highest*_left _+ *highest*_right _≥ threshold T**then **return **true endif**
13: return **false**

**Table 2:** Memory for neighborhood storage for different alphabets with adapted neighborhood lengths

indexed neighborhood alphabet	bits per character (*α*)	neighborhoods length (*L*)	total per index line (2*αL*)	relative gain compared to Σ_20 _(1 – 2*αL/*110)	
Σ_20_	5	11	110	0%	Memory
Σ_16_	4	12	96	13%	
Σ_8_	3	14	84	24%	
Σ_4_	2	19	76	31%	
Σ_2_	1	32	64	42%	

for neighborhood storage is computed with respect to sensitivity/selectivity trade-offs shown on Figure 7.

In the rest of the paper, the original alphabet of 20 amino acids is denoted by Σ_20_, where each character is encoded by 5 bits. Reduced alphabets Σ_16_, Σ_8_, Σ_4 _and Σ_2_, respectively of size 16, 8, 4 and 2, have each character encoded by 4, 3, 2 and 1 bits respectively. Those alphabets, taken from Table 2 of [15], are defined by

$$\begin{array}{lll} \Sigma_{16} & = & \{[C],[FY],[W],[ML],[IV],[G],[P],[A],\\ & & [T],[S],[N],[H],[QE],[D],[R],[K]\},\\ \Sigma_{8} & = & \{[CFYW],[MLIV],[G],[P],[ATS],\\ & & [NH],[QED],[RK]\},\\ \Sigma_{4} & = & \{[CFYW],[MLIV],[GPATS],\\ & & [NHQEDRK]\},and\\ \Sigma_{2} & = & \{[CFYWMLIV],\\ & & [GPATSNHQEDRK]\}. \end{array}$$

The main idea is to represent the neighborhoods of keys stored in the index (see Figure 3) over a reduced alphabet. Consequently, at Stage 2 of the similarity search, amino acid sequences are compared with sequences over the reduced alphabet. By an *alignment over *Σ × Σ', we understand an alignment between a sequence over Σ and a sequence over Σ'. Thus, in this paper we will consider alignments over Σ_20 _× Σ_20_, Σ_20 _× Σ_16_, Σ_20 _× Σ_8_, Σ_20 _× Σ_4 _and Σ_20 _× Σ_2_.

**Figure 5:**
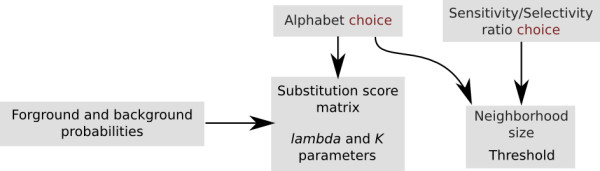
**Parameters involved in alphabet reduction**. Once an alphabet and a sensitivity/selectivity ratio are chosen, several parameters are computed. Substitution score matrix and *e*-value parameters depend only on the alphabet and the model probabilities, whereas the optimal neighborhood size and the threshold depends also on the sensitivity/selectivity level.

In the next sections, we describe how to evaluate the quality of Stage 2 and how a substantial index size reduction can be obtained by using longer neighborhoods on reduced alphabets. As presented in Figure 5, using a reduced alphabet involves several parameters that we study in the following sections. In section *Rectangular substitution score matrices*, we present substitution score matrices used for alignments over Σ_20 _× Σ_8 _and Σ_20 _× Σ_16_. We then present the computation of *e*-value to estimate the significance of alignments over reduced alphabets. The last section, *Experimental validation*, describes a practical application of reduced alphabets to real biological data.

### Stage 2 algorithm and quality

A detailed description of Stage 2 is given in Algorithm 1 (Table 1). Query and database neighborhoods of a matching key (detected during Stage 1) are compared character by character over *L *positions. During this comparison that uses substitution score matrices (lines 1 and 1), the highest scores for the left and right neighborhoods are kept (lines 1 and 1). If the sum of the highest scores exceeds a threshold T, the alignment is kept for Stage 3 (line 1), otherwise it is rejected (line 1). Note that in the offset indexing case, a random memory access is performed in order to retrieve neighborhoods left_*db *_and right_*db *_(line 1). This is not the case for the neighborhood indexing, as the neighborhoods are stored directly in the index.

The quality of Stage 2 is measured by a trade-off between its sensitivity (ability to extend true alignments) and selectivity (ability to filter out spurious seed hits). Computation of those values is described page 10.

Increasing the threshold T or decreasing the neighborhood length *L *makes Stage 2 more selective but less sensitive (faster execution at the price of worse quality results) while decreasing T or increasing *L *increases the sensitivity and decreases the selectivity (better quality results at the price of a slower execution).

### Reducing the index size by 35% without loss of quality

**Figure 6:**
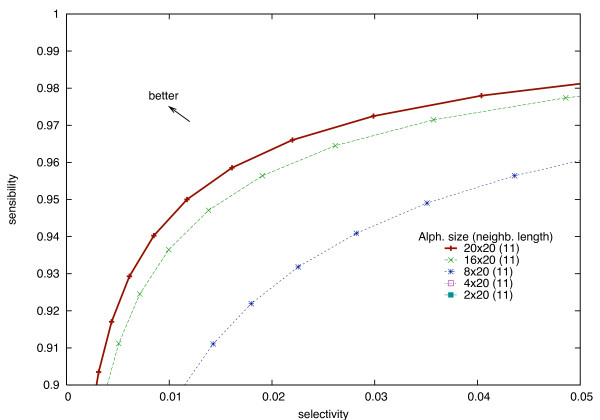
**Sensitivity/selectivity trade-off using different alphabets with a constant neighborhood length**. Sensitivity/selectivity trade-off for two neighborhoods of length 11 (other lengths give similar results). When the length is fixed, reduced alphabets provide worse results than the Σ_20 _× Σ_20 _alphabet. The curves for alphabets Σ_20 _× Σ_4 _and Σ_20 _× Σ_2_, not shown, are even worse.

As shown in Figure 6, the sensitivity/selectivity trade-off follows a convex curve. We propose here to achieve an equivalent trade-off with a reduction of the index size.

**Figure 7:**
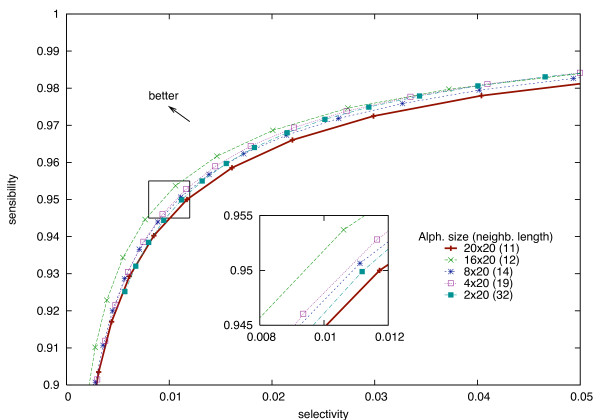
**Sensitivity/selectivity trade-off using different alphabets with adapted neighborhood lengths**. Sensitivity/selectivity trade-off for two neighborhoods with the adapted lengths of Table 2. Now all reduced alphabets are equivalent (or slightly better, due to integer rounding of the neighborhood lengths) than the original alphabet Σ_20 _× Σ_20_.

Clearly, for a fixed neighborhood length *L *(in Figure 6, 16 amino acids), the sensitivity/selectivity trade-off is always better when using the full amino acid alphabets than a reduced alphabet. This is easily explained by the fact that reducing the alphabet size decreases the alignment accuracy. In order to keep up with the sensitivity/selectivity ratio, the neighborhood length *L *should be increased. In Figure 7, all reduced alphabets, used with increased neighborhood lengths, now perform equivalently (or slightly better) than the full alphabet.

**Figure 8:**
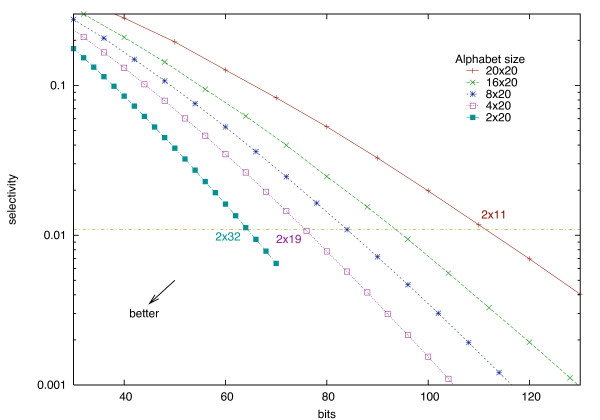
**Memory for neighborhood storage for different alphabets at a fixed sensitivity**. Memory space needed to achieve a sensitivity close to 0.95. The same quality can be achieved with 64 bits (2 neighborhoods of 32 amino acids encoded in 1 bit, sensitivity of 0.9499, selectivity of 0.0112) instead of 110 bits (2 neighborhoods of 11 amino acids encoded in 5 bits, sensitivity of 0.9500, selectivity of 0.0111). All reduced rectangular alphabets lead to smaller index sizes than the regular Σ_20 _× Σ_20 _alphabet.

Figure 8 shows the dependency, for different reduced alphabets, between the number of bits needed to store both neighborhoods (X axis) and the selectivity (Y axis), for an equivalent quality (fixed sensitivity). Those results are obtained with the use of special substitution score matrices, adapted to reduced alphabets, that are presented in the next section. Our main result is that for any given selectivity, using any of the reduced alphabets for storing neighborhoods leads to a smaller *αL *factor than for the Σ_20 _alphabet. Therefore, for a fixed memory usage, the sensitivity/selectivity trade-off is always better with a reduced alphabet than with the full Σ_20 _alphabet.

In practice, this result enables a reduction of the index size without any sacrifice in running time or in result quality. Table 2 shows the memory requirements for different alphabets. We obtain a practical reduction of 42% of the factor *αL *using the reduced alphabet Σ_2 _instead of Σ_20_. The ratio *r *on the overall index size is then reduced by 35%.

### Rectangular substitution score matrices

We designed a method for computing substitution score matrices for any pair of possibly reduced amino acid alphabets. As this method is based on the original programs of [18], we call such matrices REBLOSUM for Rectangular BLOSUM matrices. The REBLOSUM matrices for alphabets Σ_20 _× Σ_20 _are the original BLOSUM matrices. Tables 3, 4, 5 and 6 present REBLOSUM matrices for alignments over alphabets Σ_20 _× Σ_16_, Σ_20 _× Σ_8_, Σ_20 _× Σ_4 _and Σ_20 _× Σ_2 _respectively.

**Table 3:** REBLOSUM 62 matrix for alphabet Σ_20 _× Σ_16_

	*C*	*F*	*Y*	*W*	*M*	*L*	*I*	*V*	*G*	*P*	*A*	*T*	*S*	*N*	*H*	*Q*	*E*	*D*	*R*	*K*
[*C*]	**9**	-2	-2	-2	-1	-1	-1	-1	-3	-3	0	-1	-1	-3	-3	-3	-4	-3	-3	-3
[*FY*]	-2	**5**	**5**	1	0	0	-1	-1	-3	-3	-2	-2	-2	-3	0	-2	-3	-3	-2	-3
[*W*]	-2	1	2	**11**	-1	-2	-3	-3	-2	-4	-3	-2	-3	-4	-2	-2	-3	-4	-3	-3
[*ML*]	-1	0	-1	-2	**3**	**4**	1	1	-3	-3	-1	-1	-2	-3	-2	-2	-3	-3	-2	-2
[*IV*]	-1	0	-1	-3	1	1	**3**	**3**	-3	-3	-1	0	-2	-3	-3	-2	-3	-3	-3	-2
[*G*]	-3	-3	-3	-2	-3	-4	-4	-3	**6**	-2	0	-2	0	0	-2	-2	-2	-1	-2	-2
[*P*]	-3	-4	-3	-4	-2	-3	-3	-2	-2	**7**	-1	-1	-1	-2	-2	-1	-1	-1	-2	-1
[*A*]	0	-2	-2	-3	-1	-1	-1	0	0	-1	**4**	0	1	-2	-2	-1	-1	-2	-1	-1
[*T*]	-1	-2	-2	-2	-1	-1	-1	0	-2	-1	0	**5**	1	0	-2	-1	-1	-1	-1	-1
[*S*]	-1	-2	-2	-3	-1	-2	-2	-2	0	-1	1	1	**4**	1	-1	0	0	0	-1	0
[*N*]	-3	-3	-2	-4	-2	-3	-3	-3	0	-2	-2	0	1	**6**	1	0	0	1	0	0
[*H*]	-3	-1	2	-2	-2	-3	-3	-3	-2	-2	-2	-2	-1	1	**8**	0	0	-1	0	-1
[*QE*]	-3	-3	-2	-2	-1	-3	-3	-2	-2	-1	-1	-1	0	0	0	**4**	**4**	1	0	1
[*D*]	-3	-3	-3	-4	-3	-4	-3	-3	-1	-1	-2	-1	0	1	-1	0	2	**6**	-2	-1
[*R*]	-3	-3	-2	-3	-1	-2	-3	-3	-2	-2	-1	-1	-1	0	0	1	0	-2	**5**	2
[*K*]	-3	-3	-2	-3	-1	-2	-3	-2	-2	-1	-1	-1	0	0	-1	1	1	-1	2	**5**

REBLOSUM62 matrix for alphabet Σ_20 _× Σ_16_. Scores located on the "diagonal" are shown in bold.

**Table 4:** REBLOSUM 62 matrix for alphabet Σ_20 _× Σ_8_

	*C*	*F*	*Y*	*W*	*M*	*L*	*I*	*V*	*G*	*P*	*A*	*T*	*S*	*N*	*H*	*Q*	*E*	*D*	*R*	*K*
[*CF Y W *]	**4**	**4**	**4**	**5**	-1	0	-1	-1	-3	-3	-2	-2	-2	-3	0	-2	-3	-3	-3	-3
[*M LIV *]	-1	0	-1	-2	**2**	**3**	**3**	**2**	-3	-3	-1	-1	-2	-3	-3	-2	-3	-3	-2	-2
[*G*]	-3	-3	-3	-2	-3	-4	-4	-3	**6**	-2	0	-2	0	0	-2	-2	-2	-1	-2	-2
[*P *]	-3	-4	-3	-4	-2	-3	-3	-2	-2	**7**	-1	-1	-1	-2	-2	-1	-1	-1	-2	-1
[*AT S*]	-1	-2	-2	-3	-1	-2	-1	-1	0	-1	**2**	**2**	**2**	0	-1	-1	-1	-1	-1	-1
[*N H*]	-3	-2	0	-3	-2	-3	-3	-3	-1	-2	-2	-1	0	**5**	**5**	0	0	1	0	0
[*QED*]	-3	-3	-2	-3	-2	-3	-3	-3	-2	-1	-1	-1	0	0	0	**3**	**3**	**4**	0	0
[*RK*]	-3	-3	-2	-3	-1	-2	-3	-2	-2	-1	-1	-1	0	0	0	1	0	-1	**4**	**4**

REBLOSUM62 matrix for alphabet Σ_20 _× Σ_8_. Scores located on the "diagonal" are shown in bold.

**Table 5:** REBLOSUM62 matrix for alphabet Σ_20 _× Σ_4_

	*C*	*F*	*Y*	*W*	*M*	*L*	*I*	*V*	*G*	*P*	*A*	*T*	*S*	*N*	*H*	*Q*	*E*	*D*	*R*	*K*
[*CF Y W *]	**6**	**6**	**6**	**7**	-1	-1	-1	-2	-4	-5	-2	-3	-3	-4	-1	-4	-4	-5	-4	-4
[*M LIV *]	-2	0	-2	-3	**3**	**4**	**4**	**4**	-5	-4	-1	-1	-3	-5	-4	-3	-4	-5	-4	-3
[*GP AT S*]	-2	-4	-3	-4	-2	-3	-3	-2	**4**	**4**	**3**	**2**	**2**	-1	2-	-1	-1	-2	-2	-1
[*N HQEDRK*]	-5	-4	-2	-4	-3	-4	-5	-4	-2	-2	-2	-1	0	**3**	**3**	**3**	**3**	**3**	**3**	**3**

REBLOSUM62 matrix for alphabet Σ_20 _× Σ_4_. Scores located on the "diagonal" are shown in bold.

**Table 6:** REBLOSUM62 matrix for alphabet Σ_20 _× Σ_2_

	*C*	*F*	*Y*	*W*	*M*	*L*	*I*	*V*	*G*	*P*	*A*	*T*	*S*	*N*	*H*	*Q*	*E*	*D*	*R*	*K*
[*CF Y W M LIV *]	**4**	**4**	**3**	**4**	**3**	**4**	**4**	**3**	-6	-6	-2	-2	-4	-6	-4	-4	-6	-7	-5	-5
[*GP AT SN HQEDRK*]	-4	-5	-4	-6	-3	-5	-5	-4	**2**	**2**	**1**	**1**	**2**	**2**	**1**	**2**	**2**	**2**	**2**	**2**

REBLOSUM62 matrix for alphabet Σ_20 _× Σ_2_. Scores located on the "diagonal" are shown in bold.

Such matrices can be applied in any method reducing the amino acid alphabets by residue grouping. As one may be interested in using any other pair of alphabets, we additionally propose a web interface [17]. This web interface computes REBLOSUM matrices for other alphabets listed in [16] and for any custom alphabets provided by the user.

### Parameters for *e*-value computation

The *e*-value, or *expected value*, provides the expected number of alignments with a given score, when comparing a text *T *and a query *Q *of length *|T | *and *|Q| *respectively. Local alignment methods like Blast sort results by increasing *e*-value, thus reflecting their decreasing significance. In the Blast algorithm, the *e*-value of an alignment is obtained by

*e*-value = *K*·|*Q*| · |*T*| · *e*^-*λs*^,

where *s *is the score of the alignment obtained with substitution matrices. Parameters *λ *and *K *are two constants that fit the Gumbel law, computed using methods described in [19]. Table 7 provides those parameters for several REBLOSUM substitution matrices.

**Table 7:** Gumbel law parameters *λ *and *K *for different alphabets, obtained with the corresponding REBLOSUM score matrices.

alphabets	*λ*	*K*
Σ_20 _× Σ_20_	0.320	0.139
Σ_20 _× Σ_16_	0.333	0.143
Σ_20 _× Σ_8_	0.223	0.142
Σ_20 _× Σ_4_	0.212	0.128
Σ_20 _× Σ_2_	0.161	0.101

REBLOSUM62 matrix for alphabet Σ_20 _× Σ_2_. Scores located on the "diagonal" are shown in bold.

### Experimental validation

In a model where the Stage 2 alignments are ungapped, using reduced alphabets and alignments on longer neighborhoods can however affect the result quality. Indeed, the longer the neighborhoods are, the bigger is the chance to meet a gap in the sequences. More generally, the probabilities distributions used in theoretical sensitivity and selectivity computations do not truly reflect the nature of the biological sequences.

We thus validated our approach with large-scale tests on biological sequences. We set a database to be the hard-masked human chromosome 21 (UCSC Release hg18) translated according to the six possible reading frames. The query set was a set of seven archea and bacteria proteomes derived from a study of mitochondrial diseases. This set was selected for is interest toward the detection of potential insertions of mitochondrial genes in the human genome. Moreover, testing out our approach comparing such distant species represents one of the hardest application case. Indeed more typical homology searches on closer sequences is easier. Tests on such homology searches could have hidden potential issue on our approach.

The database contained 12 700 507 amino acids whereas the query was composed by 5 321 439 amino acids. Using the ssearch method [20], 650 alignments were obtained between the database and the query (maximal *e*-value: 10^-3^). This set of exhaustive *optimum *alignments was sufficient to validate our method in comparison with results obtained using different alphabets. The seed used in Stage 1 was a subset seed (see [21]), as in [9]. For the neighborhood indexing, we indexed the database using each of the alphabets Σ_20_, Σ_16_, Σ_8_, Σ_4 _and Σ_2_. We selected the neighborhood length to have a theoretical sensitivity close to 0.95 and a theoretical selectivity close to 0.01. Theoretical sensitivity and selectivity are defined according distributions presented on page 10.

This leads to indexing 2 × 11 characters for Σ_20_, 2 × 12 characters for Σ_16_, 2 × 14 characters on Σ_8_, 2 × 19 characters for Σ_4_, and 2 × 32 characters for Σ_2 _(Figure 7). The database index sizes are reported in Table 9. Using alphabet Σ_2 _instead of Σ_20 _reduces the overall index size: the ratio *r *goes from *r*_20 _= 5.58 to only *r*_2 _= 3.67, that is a 35% reduction. The initial assumption of ungapped alignments in the Stage 2 can be wrong with a neighborhood length of 2 × 32. Thus one could prefer to use the alphabet Σ_4 _with 2 × 19 characters, giving a 25% reduction of the overall index size (*r*_4 _= 4.17).

**Table 8:** Practical results for different alphabets – Quality estimations

alphabets	number of positions validating Stage 1 and Stage 2	practical selectivity	number of detected alignments	practical sensitivity
Σ_20 _× Σ_20_	2.14 * 10^6^	1.35 * 10^-3^	650 (all)	1
Σ_20 _× Σ_16_	1.39 * 10^6^	0.88 * 10^-3^	650 (all)	1
Σ_20 _× Σ_16_	0.98 * 10^6^	0.62 * 10^-3^	650 (all)	1
Σ_20 _× Σ_8_	0.62 * 10^6^	0.39 * 10^-3^	650 (all)	1
Σ_20 _× Σ_4_	3.14 * 10^6^	1.98 * 10^-3^	650 (all)	1
Σ_20 _× Σ_2_	2.93 * 10^6^	1.85 * 10^-3^	650 (all)	1

Similarity search results obtained on reduced alphabets. The number of positions tested (validating Stage 1 only and independent from the chosen alphabet) is 1.59 * 10^9^. The practical selectivity is computed dividing the number of positions validating both Stage 1 and Stage 2 by the number of positions tested.

**Table 9:** Practical results for different alphabets – Memory requirements

alphabet	α	*L*	*S*_neighborhood_	*r*
Σ_20_	5	11	1.70 * 10^9 ^bits = 212 MBytes	5.58
Σ_16_	4	12	1.52 * 10^9 ^bits = 190 MBytes	5.00
Σ_8_	3	14	1.37 * 10^9 ^bits = 171 MBytes	4.50
Σ_4_	2	19	1.27 * 10^9 ^bits = 159 MBytes	4.17
Σ_2_	1	32	1.12 * 10^9 ^bits = 140 MBytes	3.67

Database index size for neighborhood indexing on different alphabets. The first three columns are the same as in Table 2, the other two columns refer to the experience described in section "Practical results". The index size is equal to *N *× (⌈log_2 _*N*⌉ + 2*αL*), as explained in the beginning of the paper. Here *N *= 12 700 507 and ⌈log_2 _*N*⌉ = 24. The ratio *r *is against the size of the index for offset indexing, which is here *S*_offset _= *N *× ⌈log_2 _*N*⌉ = 0.30 * 10^9 ^bits = 38 MBytes.

As shown in Table 8, each of the reduced alphabets yields a practical full sensitivity, as all the 650 alignments are found in each test. Moreover, the practical selectivity, close to 10^-3^, is here better than the theoretical one (0.01).

## Conclusion

We proposed a method for reducing the index size when storing neighborhoods of seed keys in protein databases. This approach is based on reducing the alphabet of indexed data while using a longer neighborhood. We save 35% of the index size without any modification on the result quality assuming an ungapped alignment model. We provided optimal lengths for selected alphabets.

Furthermore, the proposed method requires unusual substitutions score matrices that are called REBLOSUM, for rectangular BLOSUM matrices. These matrices provide substitution scores between letters from different alphabets. We extended the computation of traditional BLOSUM matrices in order to compute REBLOSUM matrices, and adapted the computation of *λ *and *K *parameters for *e*-value estimation to reduced alphabets. We provided REBLOSUM matrices and their corresponding *λ *and *K *values for selected alphabets. Other matrices and parameters can be obtained from the website [17].

## Methods

In this section, we describe the methods we used to compute the sensitivity and selectivity of similarity search on reduced alphabets as well as the neighborhood length. We further describe the computation of REBLOSUM substitution score matrices and of the *e*-value parameter. Moreover, we explain how the threshold T is computed at Stage 2 depending on the *e*-value specified by the user. Finally, we describe how we estimated the time gain of the the neighborhood indexing over the offset indexing.

### Selectivity and sensitivity computation

The sensitivity of Stage 2 is defined by the ratio of retained "true alignments" (a "true alignment" is an alignment known to be relevant, according to a model or to a reference set like the BLOCKS database):

$$\text{sensitivity}=\frac{\text{\# successfully extended true alignments}}{\text{\# true alignments}},$$

The selectivity is defined as the ratio of retained "random alignments" (a "random alignment" means an alignment of randomly chosen amino acid pairs drawn according to an appropriate probability distribution):

$$\text{selectivity}=\frac{\text{\# successfully extended random alignments}}{\text{\# random alignments}}.$$

Note that here we focus on the behavior of Stage 2 and do not take into consideration the sensitivity/selectivity of Stage 1. In particular, in the above fractions we consider only alignments that extend a hit presumably reported at Stage 1.

The sensitivity and the selectivity of Stage 2 rely on three parameters: the alphabet choice, the neighborhood length, and the score threshold T. Given these three parameters, we applied a dynamic programming algorithm to compute the probability for the filter to retain an alignment drawn according to a given *amino acid pair distribution*. Applied to distributions of "true" and "random" alignments (foreground and background distributions, respectively), the algorithm gives a theoretical estimation of the sensitivity and the selectivity of the filter. The two distributions were the Bernoulli models (namely the expected and the observed probabilities, see below), obtained with the BLOSUM programs on the BLOCKS protein database when processing the BLOSUM-62 matrix.

In our Algorithm 1, two neighborhoods (left and right) are processed. We thus consider the sum of two maximal scores, reached in the left and right neighborhoods. The probability that this sum reaches a given threshold T at least once is computed as follows. First, we compute the probability for each neighborhood independently to reach *any *given maximal score *s *(*s *≥ 0) within the neighborhood length. Then, these two independent discrete distributions are combined to compute the T threshold requirement.

For our experiments, we calibrated the neighborhoods lengths to have a sensibility close to 0.95 and a selectivity close to 0.01, and computed related thresholds values (available of the REBLOSUM website).

### Computing REBLOSUM matrices

There are several substitution score matrices for the regular Σ_20 _× Σ_20 _alphabet, and the most common of them are matrices from the BLOSUM family [22] (BLOcks SUbstitution Matrix). They are built from the BLOCKS database of ungapped multiple alignments [23]. For a given identity level X and two amino acids *i *and *j*, the BLOSUMX score *B*_*i*, *j *_are log-likelihoods of *amino acid pair frequencies*:

$$B_{i,j}=\log\left(\frac{q_{i,j}}{p_{i}\cdot p_{j}}\right),$$

where *p*_*i*_· *p*_*j *_is the *expected *probability of aligning *i *against *j*, and *q*_*i*, *j *_is the *observed *probability of the same event in a subset of alignments of the BLOCKS database that have at least × percent of identity. (Note that the computation of *q*_*i*, *j *_takes into account different contributions provided by alignments with different identity levels.)

In our case, sequences over diferent alphabets are compared and we then have to adapt the matrix computation to compute appropriate rectangular matrices. For this purpose, the original data file (BLOCKS database version 5) was downloaded and the original programs of [18] (downloaded from [24]) were modified in order to take into account the reduced alphabet on "one side" of the matrix and compute new log-likelihood scores. Given two alphabets Σ and Σ', we compute such matrices for several identity levels X, using the log-likelihood of *groups of amino acid pair frequencies*:

$$B_{I,J}=\log\left(\frac{q_{I,J}}{p_{I}\cdot p_{J}}\right),$$

where *p*_*I*_·*p*_*J *_is the *expected *frequency of aligning any amino acids from group *I *⊆ Σ against any other amino acid from group *J *⊆ Σ', and *q*_*I*, *J *_is the *observed *frequency of the same event in a subset of alignments of the BLOCKS database that have at least × percent of identity. The recent paper [25] discovered flaws in the original BLOSUM implementation, but shows that a corrected program does not improve (and even in some cases decreases) the results quality. Therefore, we did not take the proposed modifications into account.

The website [17] proposes a selection of REBLOSUM matrices for several alphabets, as well as an interface to compute REBLOSUM matrices for any alphabet and identity level specified by the user.

### Prototype for estimating the time gain of offset indexing over neighborhood indexing

For comparing the execution time between offset indexing and neighborhood indexing, a C prototype was created. In the case of the offset indexing, the index stores positions of all seeds in an unique integer array. For each seed key, a pointer provides the first occurrence in this array. In the case of the neighborhood indexing, the index uses a (unique) structure array instead of an integer array. For each key occurrence, the structure contains the key position together with two neighborhoods.

Tests reported in Figure 4 were run on a 2 GHz PC with an AMD Opteron processor. The database size was selected so that the index fits into the main memory (4 GB) but not into the L1/L2 cache (1 MB). In those tests, the neighborhood indexing performs almost twice as fast as the offset indexing.

## Authors' contributions

All authors conceived the study. VHN created and tested the prototype estimating the time gain using a neighborhood index. LN and MG computed REBLOSUM matrices and optimal neighborhood lengths using different alphabets and created the web interface. PP performed tests on biological sequences and drafted the manuscript. DL and GK proposed the setup of the work and participated in its coordination. All authors read and approved the final manuscript.
